# Environmental Factor-Mediated Transgenerational Inheritance of *Igf2r* Hypomethylation and Pulmonary Allergic Response *via* Targeting Dendritic Cells

**DOI:** 10.3389/fimmu.2020.603831

**Published:** 2020-12-23

**Authors:** Jau-Ling Suen, Tai-Ting Wu, Yue-Hyuan Li, Chin-Lai Lee, Fu-Chen Kuo, Pearlly S. Yan, Chia-Fang Wu, Mita Tran, Chien-Jen Wang, Chih-Hsing Hung, Ming-Tsang Wu, Michael W. Y. Chan, Shau-Ku Huang

**Affiliations:** ^1^Graduate Institute of Medicine, College of Medicine, Kaohsiung Medical University, Kaohsiung, Taiwan; ^2^Research Center for Environmental Medicine, Kaohsiung Medical University, Kaohsiung, Taiwan; ^3^Department of Medical Research, Kaohsiung Medical University Hospital, Kaohsiung, Taiwan; ^4^Department of Gynecology and Obstetrics, E-Da Hospital, Kaohsiung, Taiwan; ^5^Graduate Institute of Public Health, Kaohsiung Medical University, Kaohsiung, Taiwan; ^6^School of Medicine, College of Medicine, I-Shou University, Kaohsiung, Taiwan; ^7^Division of Hematology, The Ohio State University, Columbus, OH, United States; ^8^Department of Biomedical Sciences, National Chung Cheng University, Chiayi, Taiwan; ^9^National Institute of Environmental Health Sciences, National Health Research Institutes, Zhunan, Taiwan; ^10^Department of Pediatrics, Kaohsiung Municipal Hsiao-Kang Hospital, Kaohsiung, Taiwan; ^11^Department of Community Medicine, Kaohsiung Medical University Hospital, Kaohsiung Medical University, Kaohsiung, Taiwan; ^12^State Key Laboratory of Respiratory Disease for Allergy at Shenzhen University, Shenzhen Key Laboratory of Allergy and Immunology, Shenzhen University School of Medicine, Shenzhen, China; ^13^Department of Medicine, Division of Allergy and Clinical Immunology, Johns Hopkins University School of Medicine, Baltimore, MD, United States

**Keywords:** allergic inflammation, dendritic cell, di-(2-ethylhexyl) phthalate, insulin-like growth factor 2 receptor, trans-generation

## Abstract

The developmental origin of allergic diseases has been suggested, but the molecular basis remains enigmatic. Exposure to environmental factors, such as di-(2-ethylhexyl) phthalate (DEHP; a common plasticizer), is suggested to be associated with increased childhood allergic asthma, but the causal relationship and its underlying mechanism remain unknown. This study explored the transgenerational mechanism of DEHP on allergic asthma and dendritic cell (DC) homeostasis through epigenetic modification. In a murine model, ancestral exposure of C57BL/6 mice to low-dose DEHP led to trans-generational promoter hypomethylation of the *insulin-like growth factor 2 receptor* (*Igf2r*), concomitant with enhanced Igf2r expression and increased apoptosis prominently in CD8α^+^ DCs upon ligand stimulation, with consequent reduction in their IL-12 secretion and subsequent T cell-derived IFN-γ, thereby promoting a default Th2-associated pulmonary allergic response. Increased apoptosis was also noted in circulating IGF2R^high^ human DCs. Further, in human placenta, the methylation level at the orthologous *IGF2R* promoter region was shown to be inversely correlated with the level of maternal DEHP intake. These results support the importance of ancestral phthalate exposure in conferring the trans-generational risk of allergic phenotypes, featuring hypo-methylation of the *IGF2R* gene and dysregulated DC homeostasis.

## Introduction

Accumulated epidemiological evidence has suggested that the changing environment and modern life style influence the fetal immune development and may contribute to the recent epidemic rise of allergic diseases, including asthma, and other immunological diseases as well. Notably, parental environmental exposure is thought to influence the disease susceptibility in infancy and later in life due, in part, to epigenetic modification (1–3). For example, maternal exposure to environmental pollutants, such as allergens (4), air pollution (5), and tobacco smoke (6, 7), increases the disease risk *via* its potential impact on neonatal immune system. Also, exposure to various phthalates, such as di-(2-ethylhexyl) phthalate (DEHP), a common plasticizer for a wide range of applications, including food packaging (8), poses a significant risk to the development of allergy and asthma in children (9, 10) and increased levels of maternal urinary monobenzyl phthalate metabolites during pregnancy are associated with early-onset eczema (11), while prenatal exposure to butyl benzyl phthalate (BBP) and di-n-butyl phthalate increases the risk of asthma among inner city children (12). However, much of the evidence is primarily observational and the causal relationship has yet to be established.

Allergic asthma is mediated by T helper type 2 (Th2)-dominant immune response, leading to lung inflammation and remodeling (13), and emerging evidence from various animal models has suggested that environmental risk factors may modify the epigenome and influence the disease susceptibility (14, 15), where neonatal dendritic cells (DCs) and T cells could be the target for the maternal transmission of allergic phenotypes (16, 17), furthering the support for the importance of maternal phthalate exposure on the development of allergic diseases. But, the impact of environmental exposure at physiological dosage relevant to the human exposure level has rarely been evaluated and the functional relevance of epigenetically modified genes remains to be defined, as well as the issue regarding whether it is merely an inter-generational (parental) effect or a bona fide transgenerational epigenetic inheritance remains unresolved.

In this study, we exploited the potential transgenerational effect of ancestral DEHP exposure (F0) and its likely mechanism of actions on allergic lung inflammation in offspring under conditions relevant to the route and level of exposure in humans. In the case of an environmental factor exposed pregnant female mouse (F0), the fetus (F1) and the germline of the fetus (the future F2) both can be affected *in utero*. These are considered as intergenerational (parental) effect, while the truly transgenerational effects would be found in F3 generation which is not exposed to the initial environmental chemical that has triggered the change (18). Since maternal allergy is a critical risk factor for childhood asthma (16, 19), this study explored the transgenerational effect of DEHP through maternal germ line and analyzed allergic parameters from F1 to F4 generations. The results showed that the ancestral maternal DEHP exposure promoted Th2-associated pulmonary allergic inflammation in mice from F1 to F4 generations, wherein altered DC homeostasis and pro-allergic DC functions were particularly noted. Importantly, hypomethylated *insulin-like growth factor receptor 2* (*Igf2r)* gene encoding a multifunctional protein thought to be involved in apoptosis in cardiac myocytes (20, 21), was found to be a candidate influencing the DC homeostasis and conferring the transgenerational effect of DEHP exposure on allergic response. Further, hypomethylation of the *IGF2R* gene was also noted in human placenta from a birth cohort study and its level was inversely correlated with the level of maternal DEHP intake.

## Materials and Methods

### Study Design

The objective of the study was to delineate the causal relationship between environmental DEHP exposure and the expression of allergic lung inflammation in offspring in a transgenerational design under conditions relevant to the route and level of DEHP exposure in humans. Parameters known to be important in airway allergic inflammation and Th2 responses were monitored in the control and the test groups by the use of established protocols. Epigenetic modifications, as determined by the level of DNA methylation in murine DCs and human placenta, were determined by next-generation sequencing (NGS), and candidate genes were validated by bisulfite pyrosequencing strategies. The human placenta tissues were from an on-going birth cohort study. The level of candidate gene expression and protein surface expression in DCs were then analyzed, followed by analysis of its functional outcomes, including the level of apoptosis.

### Mice and DEHP Exposure

C57BL/6 female and male mice, 6–8 weeks of age, were obtained from the National Laboratory Animal Center and maintained by the Animal Center of the Kaohsiung Medical University (KMU) in a specific pathogen-free environment. All approved animal experiments were performed according to the Institution’s guidelines (IACUC: 99032). F0 female mice were orally fed with 37 µg DEHP/kg body weight (approximately 0.74 µg DEHP/adult female mouse) in corn oil (Sigma). After the initial 10-day exposure, F0 female mice were mated with naïve C57BL/6 male mice and continued to receive DEHP until the end of study (**Supplementary Figure 1A**). The control female mice were fed with 0.07% methanol in corn oil (vehicle). To simulating the human exposure level, the selected dose of DEHP here was equivalent to human tolerable daily intake (TDI) determined by the EU Scientific Committee for Toxicity, Ecotoxicity and the Environment (22). The second to sixth litter of each dam was used to perform experiments, so the duration of exposure to F0 female mice were ranged from around 8 weeks to 20 weeks. It is important to note that only the F0 female mice were exposed to DEHP or methanol vehicle, while F1 to F4 generations and naïve male mice were not subjected to any exposure to DEHP.

### Breeding Strategy

The female F1 offspring of the vehicle or DEHP-exposed mice aged 8 weeks were bred with naïve male C57BL/6 mice to obtain F2 offspring. The F2 or F3 female mice were bred with naïve male mice to obtain F3 or F4 generation, respectively (**Supplementary Figures 1A, B**). Suckling mice were weaned from their mothers at 21 days of age. All F0 to F4 generation mice were given sterile distilled water in glass bottles and a commercial diet (Altromin Spezialfutter GmbH & Co. KG, Lage, Germany) *ad libitum*.

### Establishment and Assessment of Allergic Lung Inflammation Model

To examine the trans-generational effect of DEHP on allergic lung inflammation, a classical but relatively mild sensitization protocol was used as illustrated in **Supplementary Figure 1C**. The pups from the DEHP and vehicle control groups were intraperitoneal injection of 5 µg OVA (grade V; Sigma-Aldrich) with 0.5 mg alum (Sigma-Aldrich) on postnatal day 7 (PND7) and PND21. The pups were then exposed to aerosolized 3% OVA for 15 minutes on PND 25, 26, and 27. One day after the last OVA challenge, the cell subsets in the bronchoalveolar lavage fluids (BALFs) were determined by multi-parametric flow cytometry (LSR II; BD Biosciences). The supernatants in the BALFs were collected for cytokine measurement (ELISA, all eBiosciences). All pups in the same litter were subjected to the same experimental treatment regardless of gender unless indicated. All immunological parameters analyzed from offspring were performed at PND 28 unless indicated.

### Flow Cytometric Analysis

#### Murine Cells

The BALF cells were stained with APC-anti-CD3 (145-2C11; BD Biosciences; T cells), APC-anti-B220 (RA3-6B2; eBioscience; B cells), FITC-anti-I-A/I-E (clone M5/114.15.2; eBioscience; DCs/macrophages), PE-Cy7-anti-CD11c (N418; eBioscience), PE-anti-CCR3 (83101; R&D Systems; eosinophils) (23). For splenic DC subset analysis, splenocytes were stained with APC-anti-CD11c (N418; eBioscience), PE-anti-PDCA-1 (129c; eBioscience), CD4 (RM4-5; BD Biosciences), CD8α (53-6.7; BD Biosciences), or anti-IGF2R polyclonal Ab (R&D). Absolute number of each DC subset was calculated from total splenocytes. For dead cell detection, purified splenic cDCs were treated with ODN 1826 (10 µg/ml, InvivoGen) or anti-mouse CD40 mAb (20 µg/ml, BD Biosciences) in the presence of human Leu^27^-IGF2 (Novozymes GroPep, Thebarton, Australia) for 24 hours, and then stained with flourchrome-conjugated antibodies against CD11c, CD4, and CD8α and FITC-Annexin V and ViViD (Invitrogen). The cellular composition of BALF cells and splenic DC subsets were analyzed with a multi-parametric flow cytometer (LSR II; BD Biosciences) and FlowJo software (version 10, Tree Star, Inc, Ashland, OR).

#### Human DCs

The study was approved by the Institutional Review Board of Kaohsiung Medical University Hospital (KMUHIRB-E(I)-20170005). Peripheral blood mononuclear cells were isolated from five healthy volunteers (four males and one female, mean age: 22) after obtaining informed consents, and circulating CD1c^+^ DCs were purified using a human CD1c (BDCA-1) Dendritic cell Isolation kit (Miltenyi Biotec). The purified DCs were stimulated with or without anti-human CD40 (5C3, 20 µg/ml, BD Biosciences) followed by F(ab’)2 (polyclonal goat IgG, 40 µg/ml, eBioscience), CpG D19 (3 µM, Life Technologies Japan), and R848 (100 nM, Santa Cruz Biotechnology) for 20 h, respectively. The cells were harvested and then measured IGF2R expressions by biotin-anti-IGF2R Ab (R&D) followed by Streptavidin-APC (BD Biosciences) and BV510-anti-CD1c (L161, Biolegend). Apoptotic cells were observed by staining with FITC-Annexin V and Violet-ViViD (Invitrogen).

### Cytokines From DCs and OVA-Stimulated Splenocytes

Splenic cDCs (90–95% of purity) from immunized pups at PND 28 were positively selected using CD11c microbeads (Miltenyi Biotec) and stimulated with ODN 1826 (10 µg/ml) for 24 h. The supernatant was collected for analysis of IL-12 level (eBioscience). Splenocytes from OVA-immunized F1 pups were stimulated with OVA (10 µg/ml) for 72 h. The supernatant was collected for analysis of IFN-γ, IL-5, and IL-13 levels (eBioscience).

### Methylation Analysis by MethylCap-Seq and BisulFite Pyrosequencing

Splenic CD11c^high^ DCs from immunized F1 female offspring were subjected to MethylCap-Seq to identify the differentially methylated regions as previously described (24, 25). In brief, one microgram of sonicated DNA was incubated at room temperature on a rotator mixer in a solution containing 3.5 μg of MBD-Biotin Protein coupled to M-280 Streptavidin Dynabeads (Methyl Miner Kit, Invitrogen). Methylated DNA was enriched by collecting magnetic beads and washing three times with Bind/Wash Buffer. Library generation and 50-bp single-ended sequencing were performed on the Illumina HiSeq 2500 system according to the manufacturer’s standard protocol. All sequencing was performed at the sequencing core of the Ohio State University.

For bisulfite pyrosequencing, genomic DNAs from human placenta tissues were bisulfite-modified using EZ DNA methylation kit (Zymo Research, Orange, CA) according to the manufacturer’s protocol and subjected to bisulfite pyrosequencing analysis (26, 27). In brief, bisulfite-modified DNAs were PCR amplified using a tailed reverse primer in combination with a biotin-labeled universal primer. PCR and sequencing primers were designed using PyroMark Assay Design 2.0 (Qiagen GmbH, Hilden, Germany). Promoters of interest were PCR-amplified using RBC Sensizyme Hotstart Taq premix (RBC Bioscience, Taiwan). Five microliters of each PCR reaction was analyzed on a 1% agarose gel before pyrosequencing. Pyrosequencing was performed on the PyroMark Q24 (Qiagen) using the Pyro Gold Reagents (Qiagen) according to the manufacturer’s protocol. The methylation percentage of each cytosine was determined by the fluorescence intensity of cytosines divided by the sum of fluorescence intensity of cytosines and thymines at each CpG site. *In-vitro* methylated DNA (Millipore) was included as positive control for pyrosequencing. The sequencing data has been deposited in the Gene Expression Omnibus database (accession number: GSE102745).

Primer sequences (5’-3’) for human *IGF2R* using bisulfite pyrosequencing are listed below. Forward: GTGGGAGGGGAAATTGAG; Reverse: **gaacgccagcacatggacagc**AACTCCCTTATTCAAATAACCC; Sequencing: GGGAGGTATTTGGAGT. Primer sequence in bold denotes biotinylated universal primer sequences.

### Quantitative Reverse-Transcription Polymerase Chain Reaction

Purified splenic cDCs were treated with anti-mouse CD40 mAb (3/23, BD Biosciences) or ODN 1826 (Invivogen) for 4 h, and collected for RNA isolation *via* Purelink RNA mini kit (Invitrogen), and mRNA was converted to cDNA by SuperScript III kit (Invitrogen). Quantification of mRNA expression for *Igf2r*, normalized to glyceraldehyde-3-phosphate dehydrogenase levels (GAPDH), was performed on an ABI 7500 Sequence Detector (Applied Biosystems), using the following pairs of oligonucleotides. *Igf2r*: forward 5′-GTCATCAGCTTTGTGTGCCG-3′, reverse 5′-ACAGTACACTCCGTCGCTTG -3′; *GAPDH*: forward 5′-GTATGACTCCACTCACGGCAAAT-3′, reverse 5′- GTAGACTCCACGACATACTCAGCAC -3′. Relative mRNA level was calculated according to the 2^-ΔΔ^*CT* method.

### Human Birth Cohort and Study Design

#### Study Subjects

The study subjects were from an on-going birth cohort study in E-Da Hospital, Taiwan, since August 2009. The study was approved by the Institutional Review Board of E-Da hospital (Code: EMRP41101N (RI), EMRP31102N). Written informed consents were obtained from all study pregnant women themselves and on behalf of their study children. The detailed study design and questionnaire were comprehensively described elsewhere (28). We excluded pregnant women (1): who had history of systematic diseases such as cancer, hypertension and diabetes, chronic use of corticosteroids or immunosuppressant drugs; (2) whose age was older than 45 years; and (3) whose pregnancy was multiparous. Also, pregnant women who had physician-diagnosed allergic diseases were excluded. Eligible study women were interviewed with a standardized questionnaire and blood and one-spot urine specimens were collected and stored frozen at -70°C.

#### Study Design

One hundred and thirty-eight pregnant women who met the criteria were recruited from an on-going cohort. The levels of DEHP metabolites, including mono-(2-ethylhexyl) phthalate (MEHP), mono-(2-ethyl-5-hydroxyhexyl) phthalate (MEHHP), mono-(2-ethyl-5-oxohexyl) phthalate (MEOHP), mono-(2-ethyl-5-carboxypentyl) phthalate (MECPP), and mono-(2-carboxymethylhexyl) phthalate (MCMHP) were measured in urine samples by the method of online solid phase extraction coupled with high-performance liquid chromatography/tandem mass spectrometry with isotope dilution for quantitative detection (29), and used for calculating the respective DEHP daily intake levels at the third trimesters (Week 29 to 40) using the creatinine excretion-based model (30). Based on the calculated levels of DEHP daily intake and the availability of high-quality DNA samples from their corresponding placenta tissues, the highest 10 and the lowest 11 subjects were selected as “high” and “low” DEHP exposure group, respectively (**Supplementary Table 2**). These 21 DNA samples were subjected to bisulfite pyrosequencing.

### Detection of Human Urinary DEHP Metabolites

This study used the urine samples from the third trimester of pregnant women for the measurement of 11 phthalate metabolites, including five DEHP metabolites (MEHP, MEOHP, MEHHP, MECPP, and MCMHP), and other phthalate metabolites, including MnBP, MiBP, MEP, MBzP, MMP, and MiNP. DEHP daily intake in each pregnant woman was calculated based on the creatinine excretion-based model from the five urinary DEHP metabolites, including MEHP, MEOHP, MEHHP, MECPP, MCMHP (30). The creatinine excretion-based model was followed on the following equation:

$\text{DEHP}\;(\mu g/\text{kg}/\text{day})=[UE_{\text{sum}}(\mu \text{mol}/g\;\text{Cr})\times \text{CE}\;(g\;\text{Cr}/kg_{\text{body}\;\text{weight}}/\text{day})/(F_{\text{UE}}\times 1000)]\times MW_{\text{DEHP}}$

where UE_sum_, CE, F_UE_, and MW_DEHP_ represent the molar urinary excretion sum of the five measured urinary DEHP metabolites, creatinine excretion rate, molar fraction, and molecular weight of DEHP, respectively. Creatinine excretion rate (CE) was set to be 18 mg/kg/day for women. The values of F_UE_ are 0.059 for MEHP, 0.233 for MEHHP, 0.150 for MEOHP, 0.185 for MECPP and 0.042 for MCMHP.

### Statistical Analysis

For murine study, Mann-Whitney U tests were conducted to examine the differences between two groups and one-way ANOVA followed by Tukey’s multiple comparison tests for the differences among four groups. For human birth cohort study, median (IQR) or number (frequency) was used to describe the demographic characteristics, DEHP TDI and urinary DEHP metabolites when appropriate. ANOVA statistics for continuous variables and Fisher’s exact test for categorical variables were used to compare the difference across three groups, including high and low DEHP exposure groups, based on the DEHP daily intake, and subjects who did not participate in this study. The Spearman correlation was used to examine the correlation between maternal DEHP intake level and methylation level in DNA. Statistical tests in birth cohort study were performed by SPSS for Windows, version 13.0. (SPSS Inc., Chicago, Ill., USA); all *p*-values were two-sided and the significance was < 0.05. For human DC data, Wilcoxon signed rank test was used to compare the matched data from a single sample.

## Results

### Ancestral DEHP Exposure Promotes Allergic Phenotypes in F1-F4 Generations

To test for the transgenerational impact of DEHP exposure, F0 dams were daily exposed to human TDI dose of DEHP ten days prior to mating and during the periods of pregnancy and breast-feeding, and the offspring were then subjected to a well-established OVA–induced asthma mouse model without being further exposed to DEHP (**Supplementary Figure 1**). As exposure of F0 pregnant mice to environmental chemicals might also directly affect the next two generations (F1 and F2) through fetus and its germline, the true transgenerational phenotype without chemical exposure must be observed in F3 and beyond (31). Therefore, in the present study, the endpoint measurement in F1 was shown as intergenerational (parental) effect and those in F3 as transgenerational effect.

**Figure 1:**
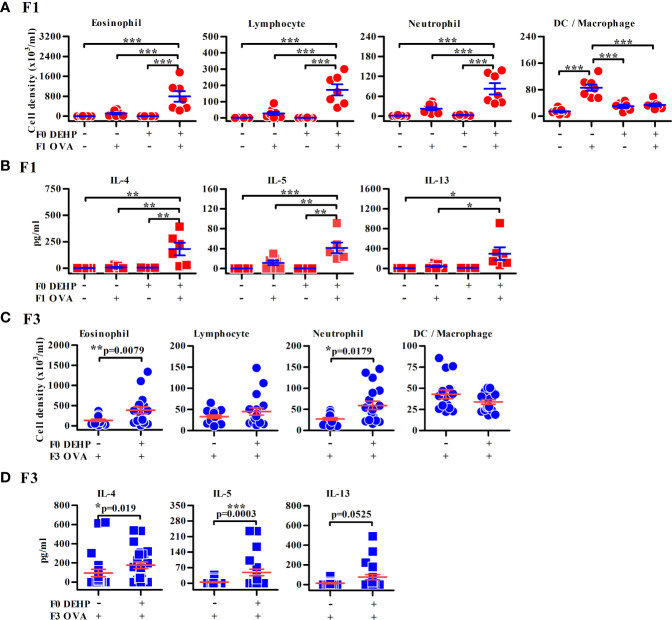
Maternal DEHP effect on allergic lung inflammation in OVA-immunized F1 and F3 offspring. F1 **(A, B)** or F3 neonates **(C, D)** from vehicle or DEHP-exposed F0 female mice were immunized with or without OVA as indicated. Then all the mice were re-challenged with OVA aerosol, as described in **Supplementary Figure 1C**. Cell subsets **(A, C)** and cytokines **(B, D)** in BALFs by flow cytometry and ELISA, respectively. Results are shown as mean ± SEM. F1: Vehicle/PBS, *n* = 7 (three dams); Vehicle/OVA, *n* = 8 (three dams); DEHP/PBS, *n* = 6 in **(A)** or 4 in **(B)** (two dams); DEHP/OVA, *n* = 7 in **(A)** or 6 in **(B)** (three dams). F3 in **(C)**: Vehicle/OVA, *n* = 15 (five dams); DEHP/OVA, *n* = 18 (five dams). F3 in **(D)**: Vehicle/OVA, *n* = 23 (six dams); DEHP/OVA, *n* = 16 (six dams). *p-value < 0.05; **p-value < 0.01; ***p-value < 0.001 by one-way ANOVA followed by Tukey’s multiple comparison test **(A, B)** or by Mann-Whitney U test **(C**, **D)**. The number of offspring (*n*) are pooled from at least two independent breeding.

As expected, elevated levels of urinary DEHP secondary metabolites, MEHHP and MEOHP, were found in DEHP-treated F0 female mice in comparison to those of vehicle control, while no evidence of exposure to DEHP in the F1 and F2 offspring (**Supplementary Figure 2**) and no apparent reproductive abnormality of F0 mothers were noted (**Supplementary Figure 3**). The F1 neonates of maternal DEHP-exposed mice developed more severe allergic lung inflammation after OVA sensitization and challenge, including predominant eosinophilia and higher levels of IL-4, IL-5, and IL-13 in the BALFs, than the control F1 neonates (**Figures 1A, B**). Similarly, enhanced airway inflammation was also noted in the F2 (**Supplementary Figures 4A, B**), F3 (**Figures 1C, D**), and F4 (**Supplementary Figures 4C, D**) progenies from DEHP-exposed F0 female mice, as compared to those found in their respective controls. In addition, the gender in offspring did not differently respond to DEHP exposure (data not shown). The enhanced levels of anti-OVA-specific IgE and of immune cell infiltration in lungs were observed in DEHP F4 neonates compared to corresponding controls (**Supplementary Figures 5A, B**). Without OVA sensitization, F1 and F2 neonates from both groups did not develop airway inflammation (**Figures 1A, B**).

### Ancestral DEHP Exposure Alters DC Homeostasis and Impairs Th1-Stimulating Activity in Immunized Young Offspring

**Figure 2:**
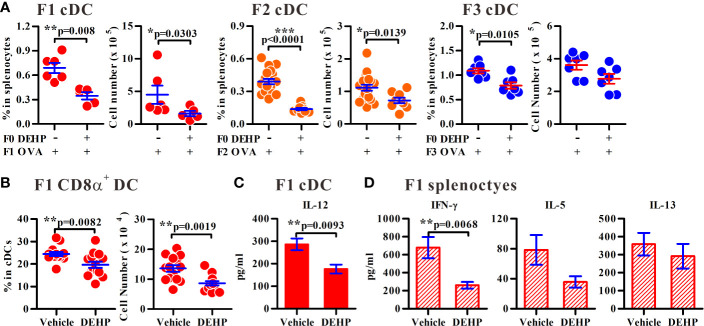
Altered homeostasis of cDCs following ancestral DEHP exposure in offspring. **(A)** The percentages or numbers of splenic cDCs in immunized F1 to F3 generations as indicated. F1: *n* = 6 (two dams) in Vehicle/OVA or *n* = 5 (two dams) in DEHP/OVA group. F2: *n* = 19 (four dams) in Vehicle/OVA or *n* = 9 (four dams) in DEHP/OVA group. F3: *n* = 7 (three dams) in each group. **(B)** The percentage and number of CD8α^+^ (CD4^-^CD8α^+^) gated on splenic cDCs in OVA-immunized F1 pups. *n* = 14 from four dams in each group. Results are shown as mean ± SEM. **(C)** Levels (mean ± SEM) of IL-12 in purified splenic cDCs from female immunized F1 pups. *n* = 8 in Vehicle or 7 in DEHP group (both from three dams). **(D)** Levels (mean ± SEM) of cytokines in OVA-stimulated splenocytes from female F1 immunized pups. *n* ≥ 6 from at least three dams in each group. *p-value < 0.05; **p-value < 0.01; ***p-value < 0.001 by Mann-Whitney U test. The number of offspring (*n*) are pooled from at least two independent breeding.

As DCs are critical in Th2-associated allergic inflammation, we sought to determine whether the homeostasis and function of splenic DCs in the immunized offspring were altered as the consequence of ancestral DEHP exposure. Interestingly, the percentages and the numbers of splenic conventional DCs (cDC; CD11c^high^PDCA-1^-^) were consistently decreased in F1 to F3 offspring of ancestral DEHP-exposed group, as compared to those noted in the corresponding controls (**Figure 2A**). Next, we examined whether the altered homeostasis of splenic cDCs contribute to the imbalanced Th1 versus Th2 activity in maternal DEHP exposed F1 offspring. Splenic CD8α^+^ cDCs and CD8α^-^ cDCs are able to promote Th1 and Th2 responses (32), respectively. In particular, CD8α^+^ cDCs are predetermined to secrete high level of IL-12 for the induction of Th1 activity (33). Thus, we analyzed the levels of splenic cDC subsets in OVA-immunized F1 young mice and found a significant loss in the absolute number of CD8α^+^ cDCs (**Figure 2B**), but not CD8α^-^ cDCs (a prominent splenic DC subset; **Supplementary Figure 6**), in the DEHP group. Further, significantly lower levels of IL-12 were found in splenic cDCs from DEHP female F1 neonates, as compared with those in control F1 mice, in response to a Toll-like receptor 9 (TLR9) agonist, oligonucleotide 1826 (ODN 1826) (**Figure 2C**). Consequently, when splenic CD4^+^ T cells from OVA-sensitized mice were analyzed, significantly lower levels of OVA-induced IFN-γ, but not IL-5 and IL-13, from the DEHP group, were found when compared to those seen in the control (**Figure 2D**). These results suggested, therefore, that ancestral DEHP exposure may disturb the homeostasis of splenic cDC subsets and lead to a default Th2 response due to impaired cDC subset and Th1 activity in offspring. It was noted that while maternal DEHP exposure enhanced Foxp3 expression level in CD4^+^CD25^+^ regulatory T cells in F1 and F2 offspring (**Supplementary Figures 7A, B**, respectively), the percentages and the suppressive function of CD4^+^CD25^+^ regulatory T cells were similar between the DEHP and the control groups (**Supplementary Figures 7C, D**).

### Epigenetic Modification of *Igf2r* is Associated With Transgenerational DEHP Effect

**Figure 3:**
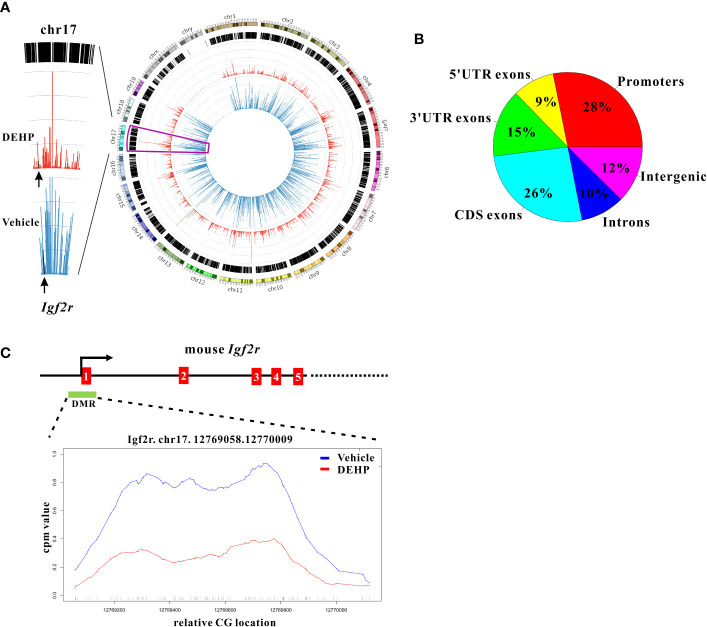
Whole genome methylation analysis and promoter methylation of *Igf2r* in cDCs. **(A)** A circos plot (right panel) showing genome-wide DNA methylation level using Methyl-Cap Seq in splenic cDCs from immunized F1 offspring born to maternal DEHP-exposed mice, compared to vehicle. (blue: vehicle; red: DEHP). The methylation level (left panel) in chromosome 17 is enlarged for better visualization, and the level of *Igf2r* is indicated by arrows. **(B)** Pie chart showing distribution of 1253 differential methylated regions (DMRs) in mm10 genome. **(C)** Schematic diagram showing genomic structure of *Igf2r* which contains a CpG island (green box, DMR) in the promoter region. Relative methylation level across the *Igf2r* promoter region in DMR (Chr17:12769058-12770009) is enlarged for better visualization.

To investigate whether altered epigenetic events in DCs could confer the dysregulated DC homeostasis and the transgenerational effect of DEHP, a genome-wide methylomic approach (MethylCap-Seq) was pursued to compare DNA methylation patterns in splenic cDCs of the F1 offspring born to maternal DEHP-exposed versus those from vehicle control groups. From this initial analysis, hypomethylation pattern was particularly noted in splenic cDCs from DEHP F1 offspring (**Figure 3A**, right panel), and a total of 1253 differential methylated regions (DMRs) were identified, the majority of which were located in the promoter and CDS regions (**Figure 3B**). Among those differentially methylated targets, *Igf2r* was selected for further analysis, as it has been shown to mediate apoptosis in myocardial cells (20) and CD8^+^ T cells (21). This prompted us to hypothesize that the level of Igf2r expression might be associated with the reduction in the number of splenic DC subset noted in DEHP progenies.

**Figure 4:**
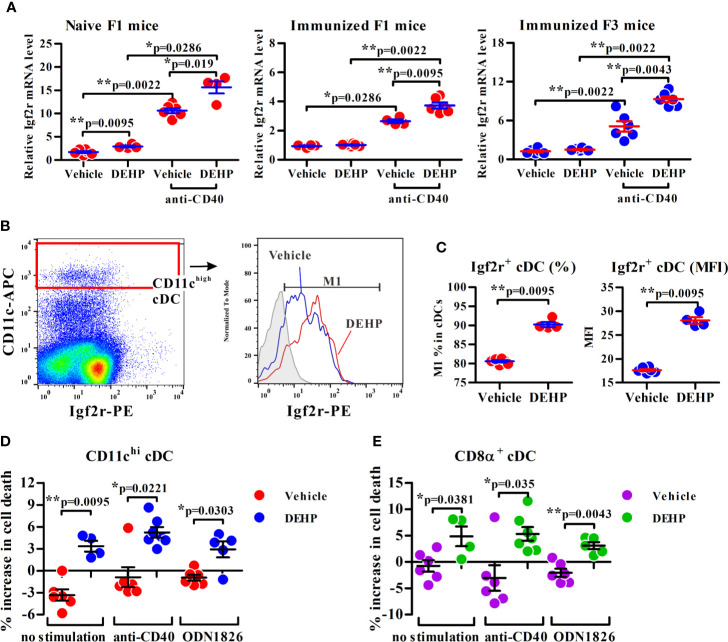
Analysis of the transmaternal DEHP effect on Igf2r expression and function in splenic cDC subsets. **(A)** Splenic cDCs from F1 or F3 pups were assessed for *Igf2r* mRNA expression by qPCR analysis. Naïve F1 mice: *n* = 6 in vehicle or 4 in DEHP group (both from two dams); Immunized F1 mice: *n* = 4 in vehicle or 6 in DEHP group (both from two dams); Immunized F3 mice: *n* = 6 in each group (both from two dams). **(B**, **C)** The frequency of Igf2r^+^ cells (M1) and expression levels of Igf2r (MFI) in gated splenic CD11c^high^ cDCs from immunized F1 offspring. Gray area in **(B)** (right panel): isotype control. MFI: mean fluorescence intensity. *n* = 6 or 4 in each group (both from two dams). One representative data set from two independent experiments. **(D**, **E)** Purified F1 cDCs treated with or without Leu^27^-IGF2 in response to anti-mouse CD40 mAb or ODN 1826 for 24 h, and assessed for apoptotic cell percentages (all Annexin V^+^ cells and ViViD^+^ cells) in CD11c^hi^ **(D)** or CD8α^+^CD11c^hi^ **(E)**-gated cells using flow cytometry. Y axis represents the increase of dead cell percentages from Leu^27^-IGF2-treated cells compared to non-treated cells. *n* = 6 or 4 in each group (both from two dams). Results are shown as mean ± SEM. *p-value < 0.05; **p-value < 0.01 by Mann-Whitney U test. The number of offspring (*n*) are pooled from two independent breeding.

Indeed, a CpG island located across the proximal promoter and exon 1 sequences of *Igf2r gene* was found to be hypomethylated in DEHP F1 cDCs as compared to vehicle control (**Figure 3A**, left panel; **Figure 3C**). Consistent with this, qPCR analysis showed that the expression of *Igf2r* was significantly upregulated in anti-mouse CD40 mAb-activated cDCs from either naïve or immunized DEHP F1 and F3 progenies; interestingly, without stimulation with anti-mouse CD40 mAbs, no difference in the level of *Igf2r* expression was found in cDCs between the DEHP and the control groups of either immunized F1 or F3 offspring (**Figure 4A**). In addition, flow cytometric analyses confirmed the increased percentages of Igf2r^+^ cells and enhanced surface expression of Igf2r in CD11c^high^ cDCs in DEHP F1 offspring in comparison with those seen in the control group (**Figures 4B, C**).

Next, the consequence of increased Igf2r expression in splenic cDCs from DEHP F1 offspring was examined by the use of a human IGF2R-specific ligand, Leu^27^-IGF2, an analog of IGF2 with Leucine to Tyrosine substitution at position 27 and interacting with both human IGF2R and mouse Igf2r (34). Results showed that while no apparent change was found in the percentage of apoptotic cells in vehicle control group, a significant, albeit not dramatic, increase in the apoptotic cell population was noted in CD11c^high^ and CD8α^+^ cDCs in response to Leu^27^-IGF2 (**Figures 4D, E**, respectively). The enhancing effect of Leu^27^-IGF2 on apoptosis was also observed in cells stimulated with either anti-mouse CD40 mAbs or a ligand for TLR9, ODN 1826 (**Figures 4D, E**). These results suggested that the transgenerational effect of DEHP on altered DC homeostasis may be, at least in part, resulted from the heritable hypomethylated CpG sites of *Igf2r* in cDCs.

### *IGF2R* Hypomethylation in Placenta is Negatively Correlated With Maternal DEHP Level

**Figure 5:**
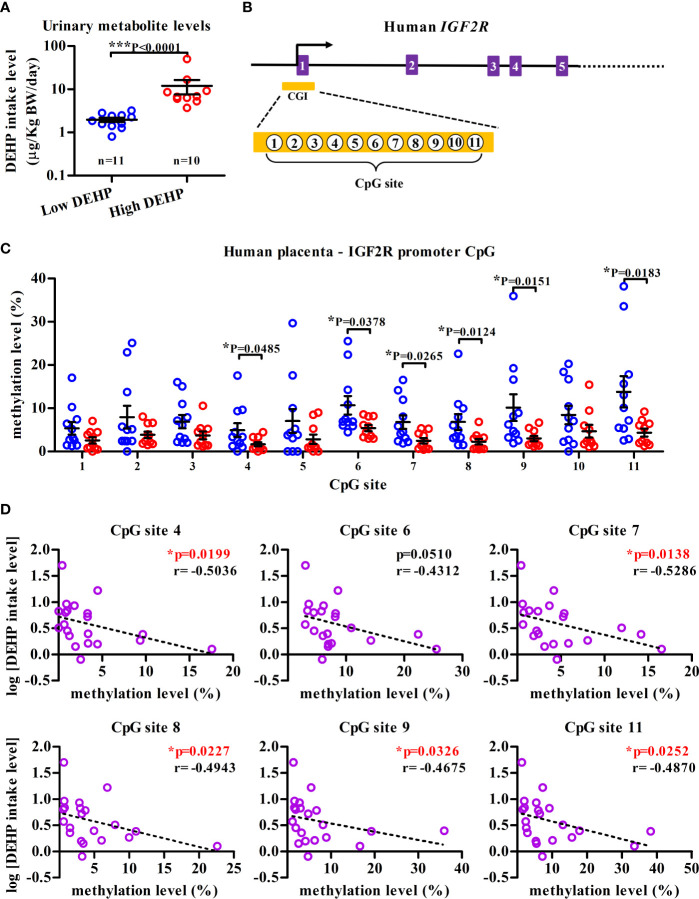
*IGF2R* hypomethylation in human placenta DNA from high-DEHP exposed mothers. **(A)** Maternal DEHP exposure levels (µg/kg body weight/day) at 38th week of gestation in average. Results are shown as mean ± SEM. **(B)** Schematic diagram illustrating partial genomic organization of human *IGF2R* and the location of a CpG island (CGI) in the promoter region. Eleven CpG sites in the CGI (Chr6:160389855-160390040) that were interrogated by bisulfite pyrosequencing are also indicated. **(C)** Placenta DNA from high- and low-DEHP exposed groups were assessed for the methylation levels of eleven CpG sites [as indicated in **(B)**] in the *IGF2R* promoter CGI using bisulfite pyrosequencing. Results are shown as mean ± SEM. *p-value < 0.05; ***p-value < 0.001, Mann-Whitney U test. **(D)** Negative correlation between maternal DEHP exposure level and the methylation level of *IGF2R* CpG sites at promoter region. *p-value < 0.05 by Spearman correlation test.

To examine whether similar epigenetic modification could be observed in human *IGF2R* locus, the methylation status of placental DNAs, serving as early-life indicators of later-life disease (35), was investigated in a panel of 21 pregnant mothers, which was dichotomized into the “high” (n=11) and the “low” (n=10) DEHP exposure groups among a total of 138 subjects from an on-going birth cohort (**Supplementary Table 2**). Except for the calculated levels of DEHP daily intake and its urinary metabolites, no significant differences in demographic characteristics were noted across three different groups, including the high, low DEHP exposure groups and a group of pregnant mothers who did not participate in this cohort (**Supplementary Table 2**). The median daily intake dose of DEHP was 6.77 µg/kg body weight/day in “high” DEHP exposure group, which was significantly higher than that in the “low” DEHP exposure group (1.86 µg/kg body weight/day; **Figure 5A** and **Supplementary Table 2**). Importantly, significantly lower methylation levels of 6 sites (site 4, 6–9, 11) among total 11 CpG sites at the promoter CpG island of *IGF2R* (hp19, Chr6:160389855-160390040, orthologue of mouse *Igf2r* promoter region; **Figure 5B**) in the high DEHP exposure group (*n* = 11) were noted, as compared with those in low DEHP exposure group (*n* = 10); (**Figure 5C**). Moreover, except for the CpG site 6, the level of methylation at the other hypomethylated CpG sites was shown to be negatively correlated with the level of DEHP exposure (**Figure 5D**).

### IGF2R Expression is Associated With Apoptosis in Human DCs

**Figure 6:**
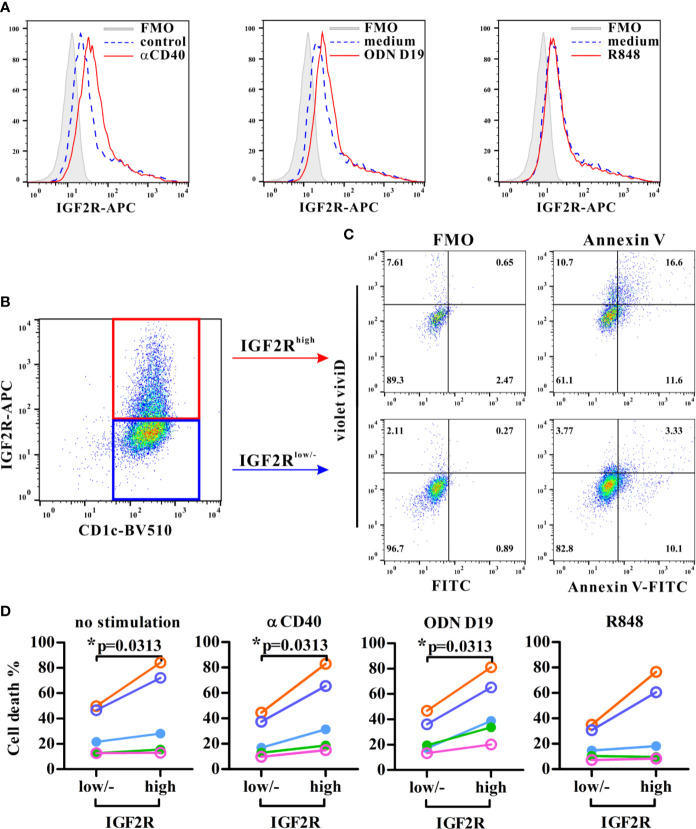
Analysis of the IGF2R expression and apoptosis in human circulating DCs. Purified human CD1c^+^ DCs were stimulated with stimuli as indicated and assessed IGF2R expression **(A)** and the percentages of apoptotic cells (all Annexin V^+^ cells and ViViD^+^ cells) within IGF2R^high^ and IGF2R^low^ subsets **(B**, **C)**. Apoptotic cells were gated against appropriate fluorescence minus one (FMO) controls. **(D)** Comparison of cell death percentages between IGF2R^high^ and IGF2R^low^ human DCs from five volunteers. *p-value < 0.05 by Wilcoxon signed rank test (one-tailed).

To determine whether human IGF2R was associated with apoptosis in DCs, flow cytometric analyses showed first that circulating CD1c^+^ DCs expressed IGF2R at resting state and in response to different stimuli (**Figure 6A**). Anti-CD40 and ODN D19 stimulation further enhanced the IGF2R expression of DCs in all five volunteers, whereas R848 agonist increased its expression level only in two subjects (**Figure 6A**). Interestingly, the frequency of cell death in IGF2R^high^ DCs was significantly higher than that in IGF2R^low^ DCs or those without detectable level of IGF2R expression, particularly when DCs were stimulated with anti-CD40 or ODN D19 (**Figures 6B–D**). These results suggested that consistent with those seen in mouse DCs, higher level of IGF2R expression on human DCs is associated with increased apoptosis in response to stimulation.

## Discussion

**Figure 7:**
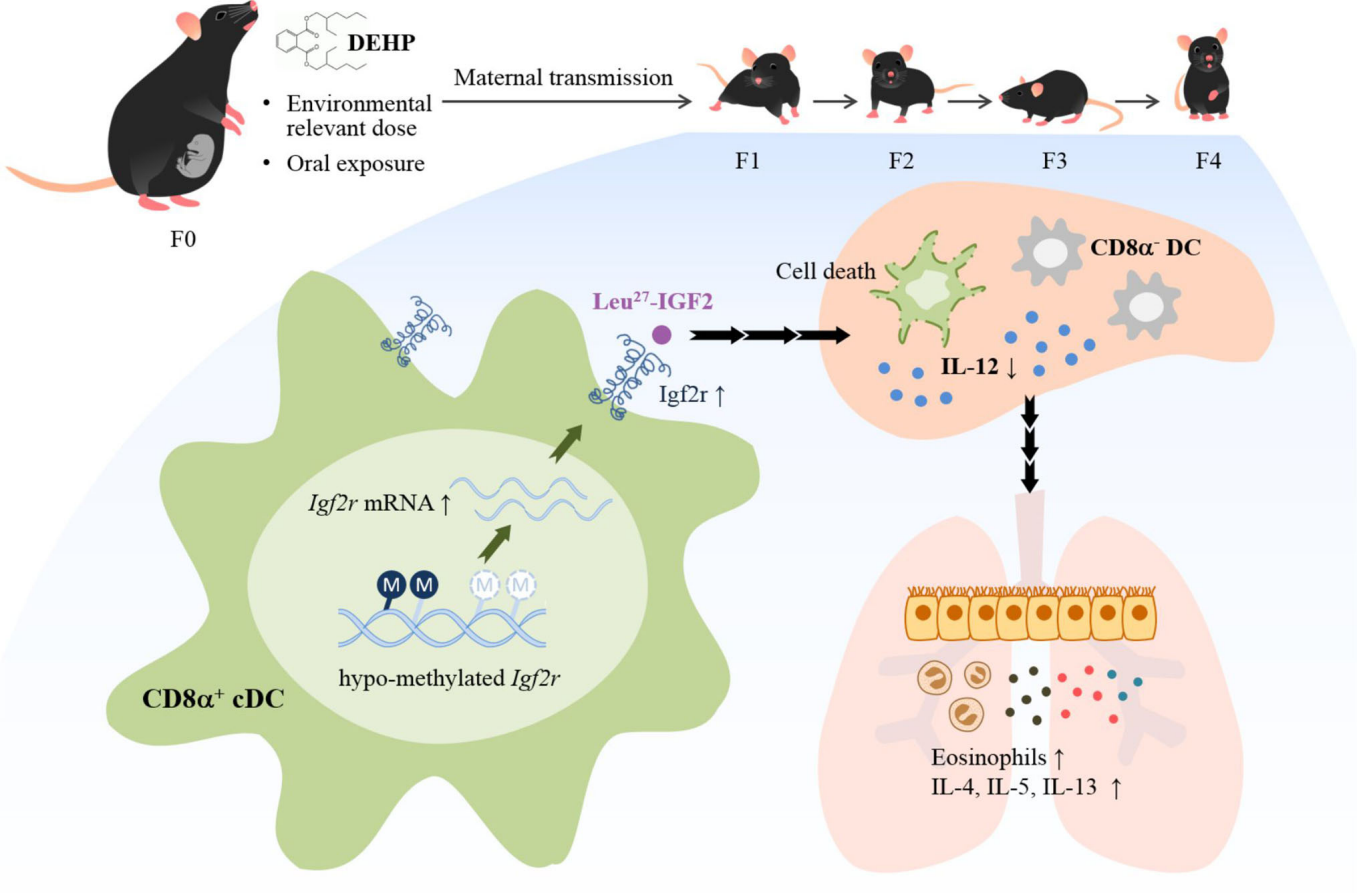
Schematic representation of DEHP transgenerational effects on allergic lung inflammation. Chronic and low-dose exposure to a common plasticizer, DEHP, may enhance allergic lung inflammation in young offspring through at least four generations. Ancestral DEHP exposure leads to trans-generational hypo-methylation of the *Igf2r* gene locus, increased expression of *Igf2r* mRNA and protein, altered homeostasis of DC subsets, and enhanced allergic lung inflammation.

Evidence is provided herein that ancestral DEHP exposure at physiologically relevant dose mimicking human exposure was shown to readily affect the developing immune system through alteration of the DC subset homeostasis, leading to enhanced allergic response for four generations. Significantly, alteration of promoter methylation of the *Igf2r* in cDCs resulted in altered homeostasis and mediated, at least in part, the transmission of allergic phenotypes (see **Figure 7** for schematic presentation). Further, analysis of human placenta DNAs from a birth cohort corroborated the results of hypomethylation in the *IGF2R* gene locus. These results thus lend a strong support for the importance of ubiquitous phthalates in modifying the epigenome, and hence the developmental origin of allergic diseases.

In humans, DEHP exposure *via* dietary sources is unavoidable due to its ubiquitous distribution (8). In the present study, to mimic human exposure way and period, the F0 female mice were orally exposed to DEHP daily starting from 6–8 weeks to around 20 weeks of age. During this exposure period, the F0 female mice were pregnant, lactated, and delivered several litters. The second to sixth litters were used to perform experiments because the first litter was not usually alive due to lack of experience for caring neonates by mother mice. The DEHP effect could be observed starting from the second litter of each dam; therefore, around 8-week exposure is required to have transmaternal DEHP effects in this experimental setting. Another important issue is the DEHP metabolite distribution in F0 and offspring. DEHP is known to be rapidly metabolized into MEHP, which is further metabolized into MEHHP and MEOHP (36). The half-life of DEHP is estimated to be about 12 h in humans (36). Due to the short half-life and fast excretion, MEHHP and MEOHP in urines can be used as biomarkers to reflect the DEHP exposure over time. In the present study, urinary levels of MEHHP and MEOHP were indeed only detectable in F0 female mice, but not in F1 and F2 offspring irrespective of gender and age (**Supplementary Figure 2**). Our results suggest that, although DEHP is metabolized quickly *in vivo* (36), chronic and environmentally relevant dose exposure may still impact the immune responses in next generations.

As a corollary, while no functional relevance was demonstrated, Li et al. showed that prenatal exposure to DEHP at around human TDI level could affect DNA methylation of imprinted genes not only in F1 fetal mouse germ cells and oocytes, but also in F2 oocytes (37). Also, the inter-generational (parental) effect of BBP on allergic asthma has been observed (17), in which maternal BBP exposure was shown to promote airway inflammation over 2 generations and hypermethylation of the gene encoding GATA-3 repressor zinc finger protein 1 (Zfpm1) was suggested as a candidate associated with Th2-driven allergic asthma in murine F1 neonatal splenic CD4^+^ T cells; however, the possibility that it is due to the effect of maternal BBP on F1 fetus and F2 germline cannot be ruled out (18, 31) and the functional consequences in allergic responses are unclear. In contrast, our study of splenic DCs revealed primarily a hypomethylation pattern and hypomethylated *Igf2r* gene being functionally critical in controlling DC subset homeostasis. Collectively, our results and those of others further support the importance of maternal phthalate exposure on the development of allergic diseases via, in part, epigenome modification.

Contrary to our study, maternal exposure to DEHP in BALB/c mice at much higher doses, 3.88 or 4.78 mg/kg body weight/day during pregnancy or lactation, respectively, inhibited OVA-induced allergic lung inflammation in the F1 neonates of maternal DEHP-exposed group as compared to those seen in the un-exposed group (38). The discrepancy could be due to the difference in the mouse strain used, the exposure time period and dosage (37 µg vs. around 4000 µg/kg body weight/day), the latter of which appears to be significant, as the transgenerational effect of DEHP noted in our study was under the condition within the range of typical human exposure ranging from 3 to 30 μg/kg body weight per day in the general population (39), which was significantly lower than those typically used in the toxicological studies (15). Also, hypomethylation of *Igf2r* gene and its impact on the expression in splenic cDCs were found in F1 and F3 generations, suggesting a likely transgenerational event involving inheritable epigenetic modification.

It was noted that at the resting state, hypomethylation of the *Igf2r* promoter CpG island did not appear to influence the expression level of *Igf2r*, but its functional impact on the level of expression and apoptosis became evident upon stimulation (see **Figure 4**), suggesting its *de novo* transcriptional effect on the *Igf2r* gene expression. Within the region encompassing the promoter CpG island, several transcriptional factor binding sites were predicted, whose activities could be influenced by the methylation status of *Igf2r*’s promoter CpG island. Further, it is noteworthy to note that, *Igf2r* is an imprinting gene which contains another intronic CpG island, known as imprinted control region (ICR), for the transcription control of *Igf2r* expression, *via* expression of a long non-coding RNA (PMID:23444351, 11845212). While the detailed mechanisms through which the promoter CpG island influences trans- and/or cis-regulated transcription remains unclear, our current results provide a foundation needed for further mechanistic investigation. Also, investigation into the potential additional immune modulatory effect as the consequence of increased Igf2r expression is warranted.

It is noted that the CD8α^+^ DC subset, appearing from day 6 onward after birth in the spleen, is the primary IL-12-producing DC subsets and known to skew the early Th2 bias into Th1 immunity in neonates (40). The functional deficiency in this CD8α^+^ DC subset noted in the offspring born to DEHP-exposed mice may thus be critical in promoting the enhanced Th2-associated allergic responses. A likely alternative, but not mutually exclusive, explanation is that the pro-allergic function of DCs in the offspring from DEHP-exposed mice may be associated with the upregulation of Igf2r expression or derived from an as yet unidentified pathway in the high Igf2r-expressing DC subsets. Consistent with the findings in mouse DCs, higher levels of apoptosis were also noted in human IGF2R^high^ DCs as compared to those of IGF2R^low^ DCs or those being negative for IGF2R expression. As a corollary, overexpression of IGF2R has been shown to negatively impact on cell growth (41), and IGF2R down-regulation results in decreased sensitivity of hypoxia- and TNF-induced apoptosis in cardiac myocytes, which was associated with reduced levels of cathepsins (20, 42). Therefore, it is likely that up-regulation of IGF2R may trigger activation of cathepsins and induce apoptosis of DCs upon stimulation. While the detailed mechanism through which Igf2r regulates cDC homeostasis and, perhaps, other cell types awaits further investigation, there is a strong possibility that the IGF2-IGF2R axis may serve as a common regulator in maintaining the cellular homeostasis, and dysregulation of its expression and activity may result in adverse immune response, such as allergic responses.

In the present study, the gender in offspring did not respond differently to transgenerational DEHP effect, including allergic parameters in BALFs, *Igf2r* mRNA and protein expression in DCs. One possible explanation for no observing gender difference is that the promoter region controlling *Igf2r* does not contain any androgen-receptor or estrogen-receptor responsive elements as deremined by JASPAR 2018 (43) in the mouse genome (mm10). Another possible reason is that the age of offspring for analyzing allergic phenotypes and DC homeostasis was 4-week-old of age, which is about 6-month-old, the weaned age in humans (44), suggesting that gender-specific hormone regulation has not yet been noted. As DC development and function is regulated by estrogen (45), it is worthy to analyzing the transgenerational effect of DEHP on those come to the age of puberty, adolescence and adulthood, including the methylation status of *Igf2r* during each time frame and the impact of gender-specific hormones. Our current design and results provide the needed foundation to elucidate the gender differences in the prevalence of wheezing and asthma in humans (46) and the effect of multiple exposure to environmental endocrine disruptors in real life.

The limitation in this study is that airway hyper-responsiveness or resistance was not performed using invasive or non-invasive means. Therefore, the conclusion was made cautiously as the mouse model of “pulmonary allergic inflammation” was used throughout. While allergic inflammation alone may not necessarily indicate airway hyperreactivity (47), the parameters known to be important in airway allergic inflammation and Th2 responses were monitored, which are known to be associated with asthma and critical phenotypes.

In summary, the current study provides a plausible epigenetic explanation and evidence supporting a causal relationship between transgenerational DEHP exposure and allergic lung inflammation in offspring. It also provides a rational basis for re-evaluating the current risk assessment of phthalate exposure and for the development of effective treatment and preventive strategies.

## Data Availability Statemen

NGS data were deposited to the Gene Expression Omnibus database (accession number: GSE102745).

## Ethics Statement

The studies involving human participants were reviewed and approved by The Institutional Review Board of E-Da hospital. The patients/participants provided their written informed consent to participate in this study. The animal study was reviewed and approved by The Institutional Animal Care and Use Committee of Kaohsiung Medical University.

## Author Contributions

J-LS, MC, M-TW, and S-KH conceived and designed the study. T-TW, Y-HL, C-LL, MT, C-JW, and PY performed the experiments and analyzed the data. F-CK and C-HH recruited pregnant women and collected samples. C-FW and M-TW analyzed human data. J-LS, MC, and S-KH analyzed the data and wrote the paper. J-LS, MC, M-TW, and S-KH revised the paper. All authors contributed to the article and approved the submitted version.

## Funding

This work was supported by the fund National Health Research Institutes (NHRI-102A1-PDCO-03010201, EOPP10-014, EOSP07-014) (to S-KH), Kaohsiung Medical University Aim for the Top Universities Grant (KMU-TP104A05, KMU-TP105A02) (to J-LS), Ministry of Science and Technology grant (MOST 105-2320-B-037-015, MOST 106-2314-B-037-072, MOST 107-2314-B-037-003, MOST 108-2314-B-037-001, MOST 108-2320-B-037-010) (to J-LS), Kaohsiung Medical University “The Talent Plan” (105KMUOR04), Shenzhen Science and Technology Peacock Team Project (KQTD20170331145453160) (to S-KH), Kaohsiung Medical University Research Center Grant (KMU-TC108A01), and Research Center for Environmental Medicine in Kaohsiung Medical University from the Featured Areas Research Center Program within the framework of the Higher Education Sprout Project by the Ministry of Education (MOE) in Taiwan. Ministry of Science and Technology grant (106-2314-B-194-002) (to MC), National Institute of Health (P30CA16058, The Ohio State University Comprehensive Cancer Center), National Institute of Health (R50CA211524) (to PY).

## Conflict of Interest

The authors declare that the research was conducted in the absence of any commercial or financial relationships that could be construed as a potential conflict of interest.

## Abbreviations

BALF, Bronchoalveolar lavage fluid; BBP, Butyl benzyl phthalate; DC, Dendritic cell; DEHP, Di-(2-ethylhexyl) phthalate; MEHP, mono-(2-ethylhexyl) phthalate; MEHHP, mono-(2-ethyl-5-hydroxyhexyl) phthalate; MEOHP, mono-(2-ethyl-5-oxohexyl) phthalate; Igf2r/IGF2R, Insulin-like growth factor 2 receptor; TDI, Tolerable daily intake.
