# Supplementary material for: Environmental Factor-Mediated Transgenerational Inheritance of Igf2r Hypomethylation and Pulmonary Allergic Response via Targeting Dendritic Cells
Source: Front Immunol. 2020 Dec 23;11:603831. doi: 10.3389/fimmu.2020.603831 (PMC7786300; doi:10.3389/fimmu.2020.603831)
Supplement: Supplementary file 1 [file DataSheet_1.pdf]

Supplementary Material

SUPPLEMENTARY MATERIALS AND METHODS

Detection of murine urinary DEHP metabolites

Two common phthalate monoester metabolites MEHHP and MEOHP were measured using liquid chromatography-electrospray ionization-tandem mass spectrometry (AB sciex; API400Q LC-MS/MS) in murine urine as described previously (1). One-hundred μl of each pooled urine sample as indicated was subjected for analysis. The limits of detection (LOD) for both MEHHP and MEOHP were 0.1 ng/ml. The measurements for DEHP metabolites were performed at a central analytical laboratory, certified by an international laboratory comparison program (G-EQUAS 52), at the National Health Research Institute in Taiwan. Urinary creatinine level was measured by the spectrophotometric method at the central laboratory of Kaohsiung Medical University Hospital in Taiwan. Creatinine-corrected adjustment for each sample was used to evaluate the DEHP exposure levels in female F0 mice and their offspring.

Regulatory T cell suppressive assay

The function of regulatory $CD4^+$ T cells (Tregs) was examined using a 5-carboxyfluorescein diacetate succinimide ester (CFSE) inhibition assay. $CD4^+CD25^+$ Tregs from offspring and $CD4^+$ T cells from naïve syngeneic mice were purified with monoclonal Ab-coated magnetic beads as per manufacturer's instructions (Miltenyi Biotec, Sunnyvale, CA). CFSE-labeled $CD4^+$ T cells (Tresp) (5×10^4/well) were co-cultured with Tregs at different ratios for 3 days. For stimulation, anti-CD3 and anti-CD28-coated beads (Dynal, Oslo, Norway) were added at a bead-to-cell ratio of 1:3. After 3 days, the cells were harvested and stained with 7-AAD (eBioscience) and analyzed by a LSR II (BD biosciences). Acquired data were analyzed using FlowJo software (version 10). To compare the suppressive capacities of Tregs in different conditions, percentages of proliferation at different Tresp-Treg ratios were calculated relative to the maximal proliferation (M1 gate) of responder cells (ratio 1:0).

SUPPLEMENTARY TABLES

Table S1 Pathway analysis from a partial gene list of imprinted genes with differential methylated regions.

Term of Pathways	*p*-value	Genes
Regulation of gene expression by genetic imprinting	0.04	*Igf2r*, *Mecp2*, *Kcnq1ot1*, *Kcnq1*
Genetic imprinting	0.05	*Cdkn1c*, *Nespas*, *Gnas*

Table S2 Demographic characteristics and urinary DEHP metabolites of participant and non-participant mothers and their newborns.

Variables	High exposure	Low exposure	Other	*p*-value[b]
n	10	11	117	
		Median (IQR) or *n* (%)		
Mother				
Pre-pregnancy BMI[a] (kg/m^2)	21.3 (19.4-24.4)	21.1 (20.3-24.0)	21.3 (19.2-25.0)	0.79
Weight gain (kg)	9.5 (6.6-17.0)	9.5 (7.8-12.3)	8.0 (6.0-10.9)	0.47
Age (years)				
≦30	5 (50.0)	3 (27.3)	58 (49.6)	0.41
>30	5 (50.0)	8 (72.7)	59 (50.4)	
Parity				
=1	5 (50.0)	2 (18.2)	50 (42.7)	0.23
≥2	5 (50.0)	9 (81.8)	67 (57.3)	
Education level[c]				
< college	5 (55.6)	3 (27.3)	52 (46.4)	0.45
≥college	4 (44.4)	8 (72.7)	60 (53.6)	
Cigarette smoking[d]				
Yes	0	1 (9.1)	6 (5.6)	0.69
No	8 (100.0)	10 (90.9)	102 (94.4)	

Calculated DEHP intake (µg/kg body weight/day)	6.77 (6.06-9.20)	1.86 (1.41-2.49)	3.17 (2.01-4.65)[e]	<0.001
DEHP metabolites (µg/g cr)				
MEHP	18.85 (7.34-31.94)	0.54 (0.36-6.90)	6.29 (2.65-13.56)[e]	0.08
MEHHP	53.76 (34.06-72.33)	14.43 (9.31-20.58)	24.23 (14.42-34.70)[e]	<0.001
MEOHP	45.07 (31.95-61.08)	11.73 (9.62-15.96)	18.61 (12.53-26.38)[e]	<0.001
MECPP	71.48 (57.68-100.03)	22.38 (12.72-25.49)	30.33 (18.23-47.34)[e]	0.001
MCMHP	16.21 (12.98-19.69)	4.72 (2.74-7.31)	6.46 (4.56-10.28)[e]	<0.001
Newborn				
Gender				
Boy	6 (60.0)	9 (81.8)	55 (47.0)	0.07
Girl	4 (40.0)	2 (18.2)	62 (53.0)	
Gestation age (weeks)	40.0 (38.0-40.0)	39.0 (38.0-40.0)	39.0 (38.0-40.0)	0.48
Birth height (cm)	50.0 (48.5-51.0)	50.0 (49.0-51.0)	50.0 (48.5-51.0)	0.54
Birth weight (g)	2997.5 (2800.0-3275.0)	3080.0 (2940.0-3320.0)	3070.0 (2800.5-3300.0)	0.73
Birth head circumference (cm)	33.0 (32.0-35.0)	34.0 (33.0-35.0)	33.5 (33.0-34.5)[f]	0.70

Birth chest circumference (cm)	32.0 (31.0-33.0)	32.5 (31.0-33.5)	32.5 (31.5-34.0)[f]	0.24

Abbreviation: BMI = body mass index; cr = creatinine; DEHP = di-(2-ethylhexyl) phthalate; IQR = interquartile range; MCMHP = mono-(2-carboxymethylhexyl) phthalate; MECPP = mono-(2-ethyl-5-carboxypentyl) phthalate; MEHHP = mono-(2-ethyl-5-hydroxyhexyl) phthalate; MEHP = mono-(2-ethylhexyl) phthalate; MEOHP = mono-(2-ethyl-5-oxohexyl) phthalate.

[a]Pre-pregnancy BMI: Body Mass Index before pregnancy

[b]*p*-value: Fisher's exact test for categorical variable and ANOVA test for continuous variable

[c]Missing data, *n* = 6 (1, 0, 5).

[d]Missing data, *n* = 11 (2, 0, 9).

[e]Missing data, *n* = 14.

[f]Missing data, *n* = 1.

SUPPLEMENTARY FIGURES

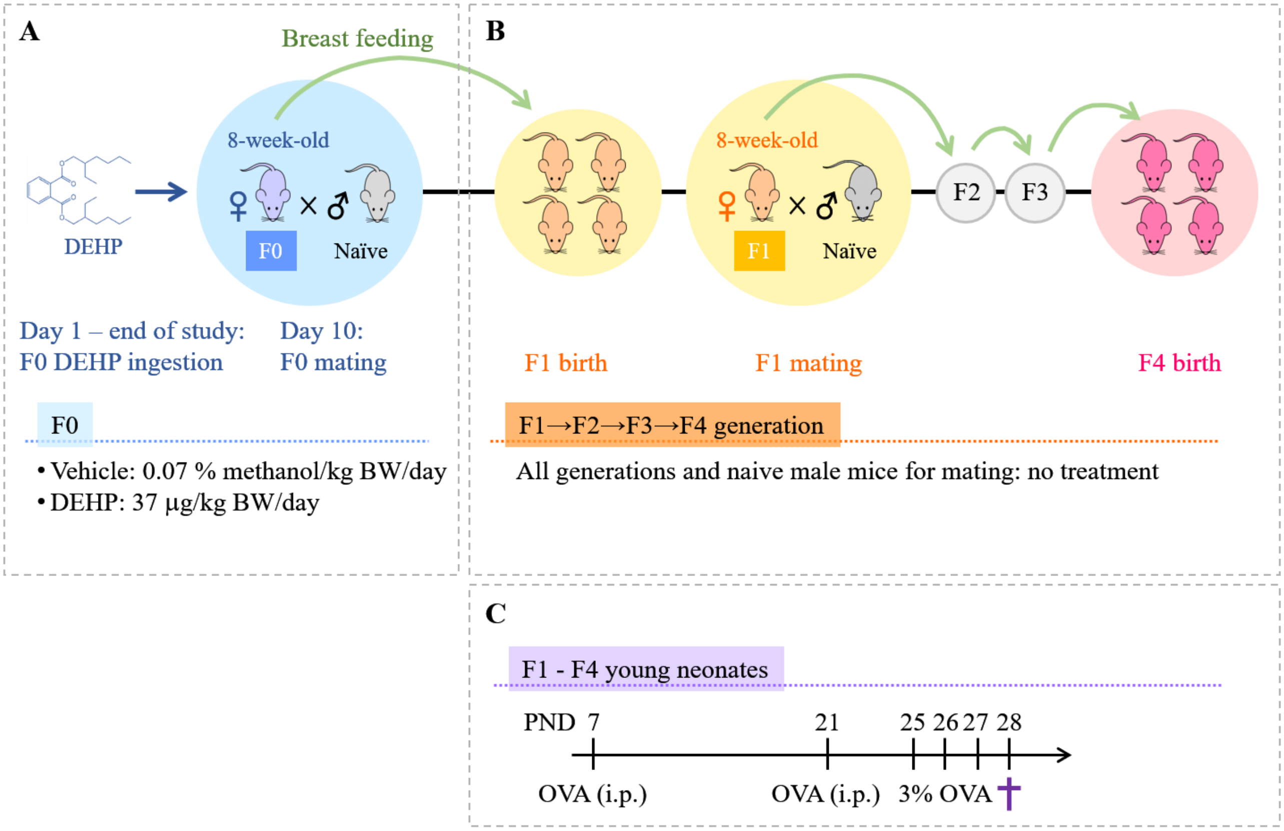

Supplementary Figure 1. Schematic depiction of experimental protocols. **(A)** F0 female C57BL/6 mice were orally fed with either DEHP (DEHP mother) or vehicle alone (control mother) until the end of study. After 10-day oral feeding, the F0 female mice were mated with naïve syngeneic male mice to generate F1 offspring. **(B)** F1, F2, or F3 female offspring from two groups were mated with naïve male mice to generate F2, F3 or F4 offspring, respectively. F1 to F3 female offspring and naïve male mice for mating were not subjected to any oral treatment. **(C)** Allergen immunization and challenge protocol for pups. All pups in the same litter were subjected to the same experimental treatment regardless of gender unless indicated. All immunological parameters analyzed from offspring were performed at postnatal day (PND) 28. OVA, ovalbumin; i.p., intraperitoneal injection.

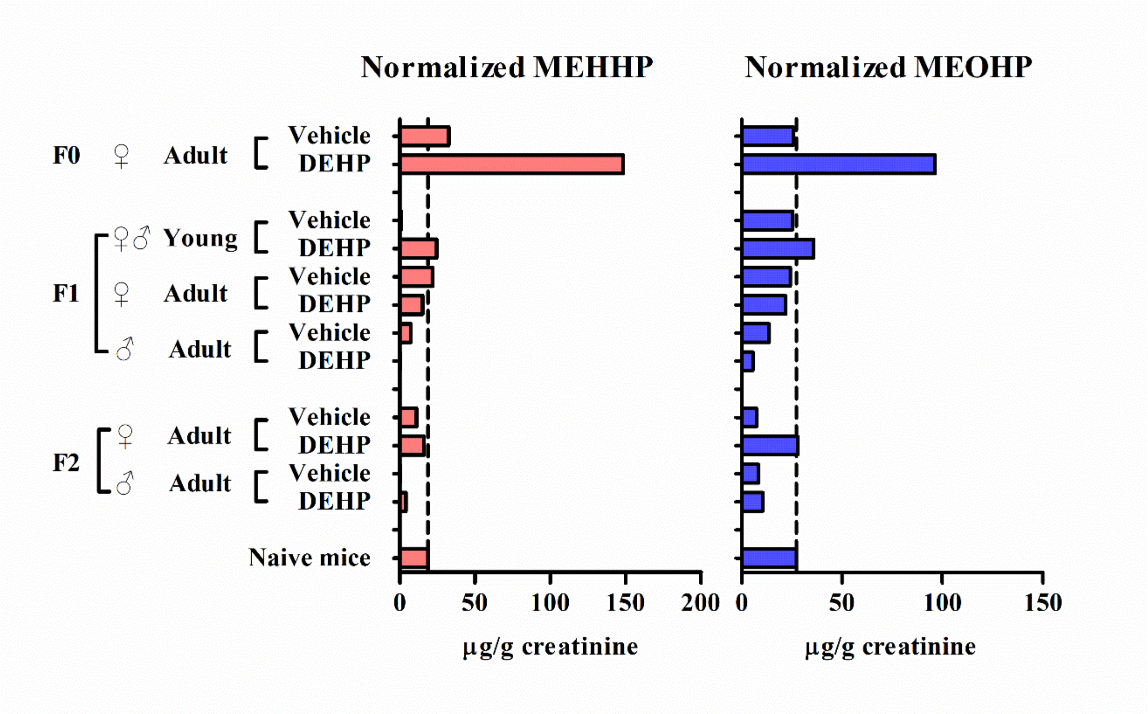

Supplementary Figure 2. Detection of DEHP metabolites in murine urine. Due to the limited urine in mice, each urine sample was pooled from various numbers of mice as indicated (*n* = 2-4 in each group). The levels of DEHP metabolites MEHHP (left) and MEOHP (right) are normalized to urinary creatinine levels of each samples. MEHHP = mono-(2-ethyl-5-hydroxyhexyl) phthalate; MEOHP = mono-(2-ethyl-5-oxohexyl) phthalate.

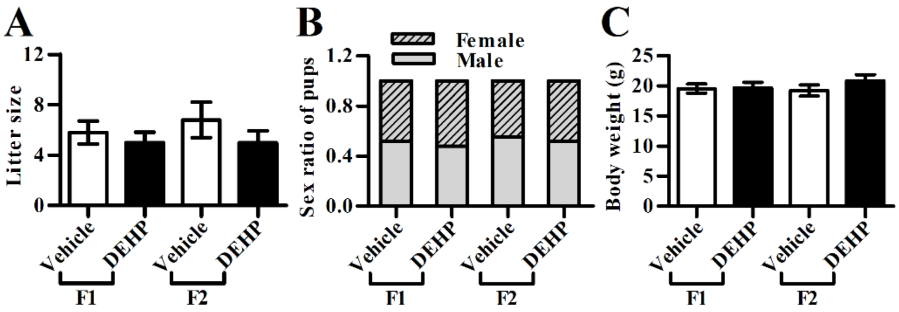

Supplementary Figure 3. Reproduction performance of C57BL/6 mothers following daily oral treatment to DEHP. Litter size **(A)** and sex ratio **(B)** of F1 and F2 offspring. Five litters from 3 dams in each group. **(C)** Body weight of F1 and F2 offspring regardless of sex at the age of 9 week-old. The results are presented as mean ± SEM.

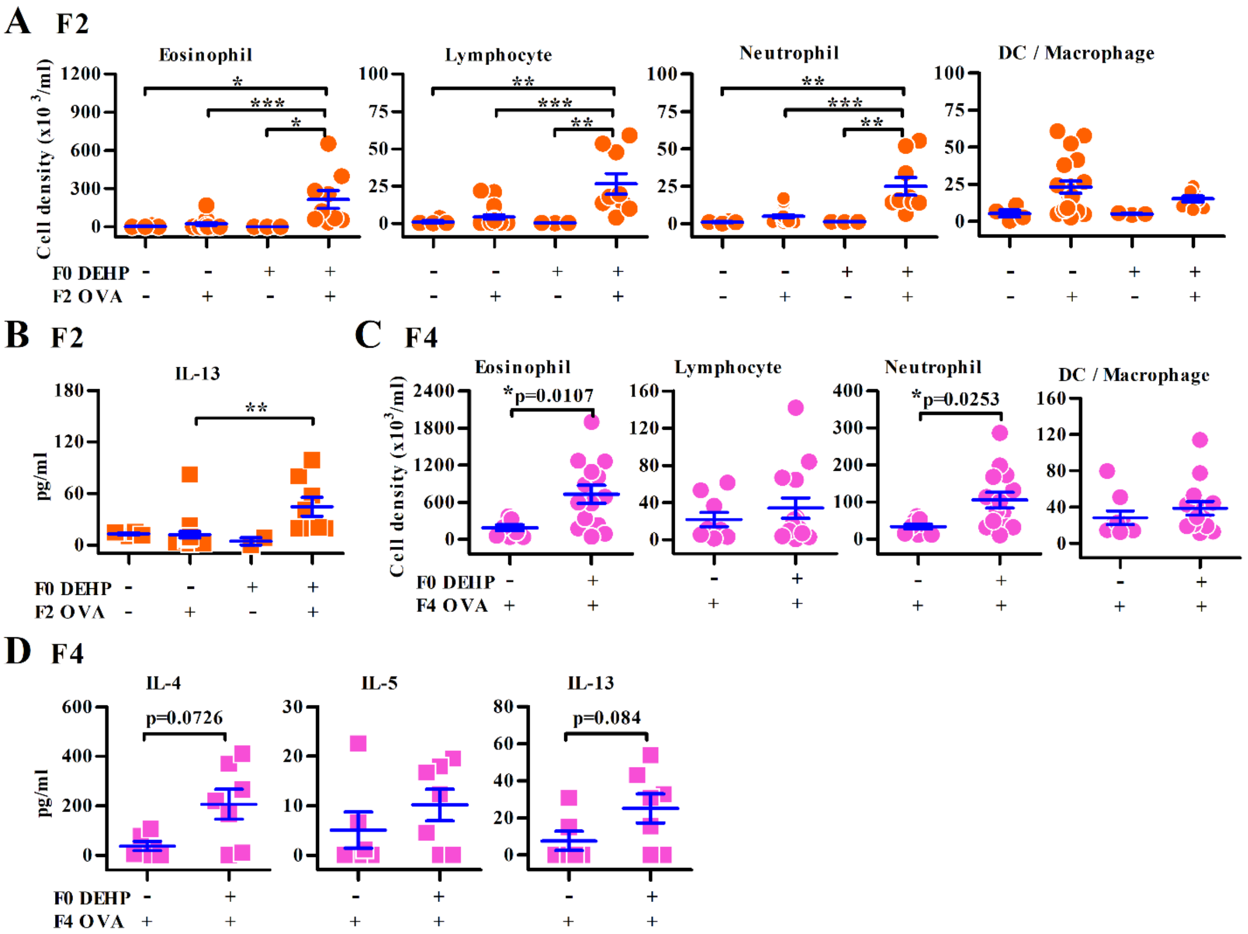

Supplementary Figure 4. Maternal DEHP effect on allergic lung inflammation in OVA-immunized F2 and F4 offspring. F2 **(A, B)** or F4 neonates **(C, D)** from vehicle or DEHP-exposed F0 female mice were immunized with or without OVA as indicated. Then all the mice were re-challenged with OVA aerosol, as described in **Supplementary Figure 1C**. Cell subsets **(A, C)** and cytokines **(B, D)** in BALFs by flow cytometry and ELISA, respectively. Results are shown as mean ± SEM. n = 3-20 animals each group in the F2 offspring (≥ 2 dams). n = 6-14 animals each group in the F4 offspring (≥ 2 dams). *p-value < 0.05; **p-value < 0.01; ***p-value < 0.001 by one-way ANOVA followed by Tukey's multiple comparison test **(A, B)** or by Mann-Whitney U test **(C, D)**. The number of offspring (n) are pooled from at least two independent breeding.

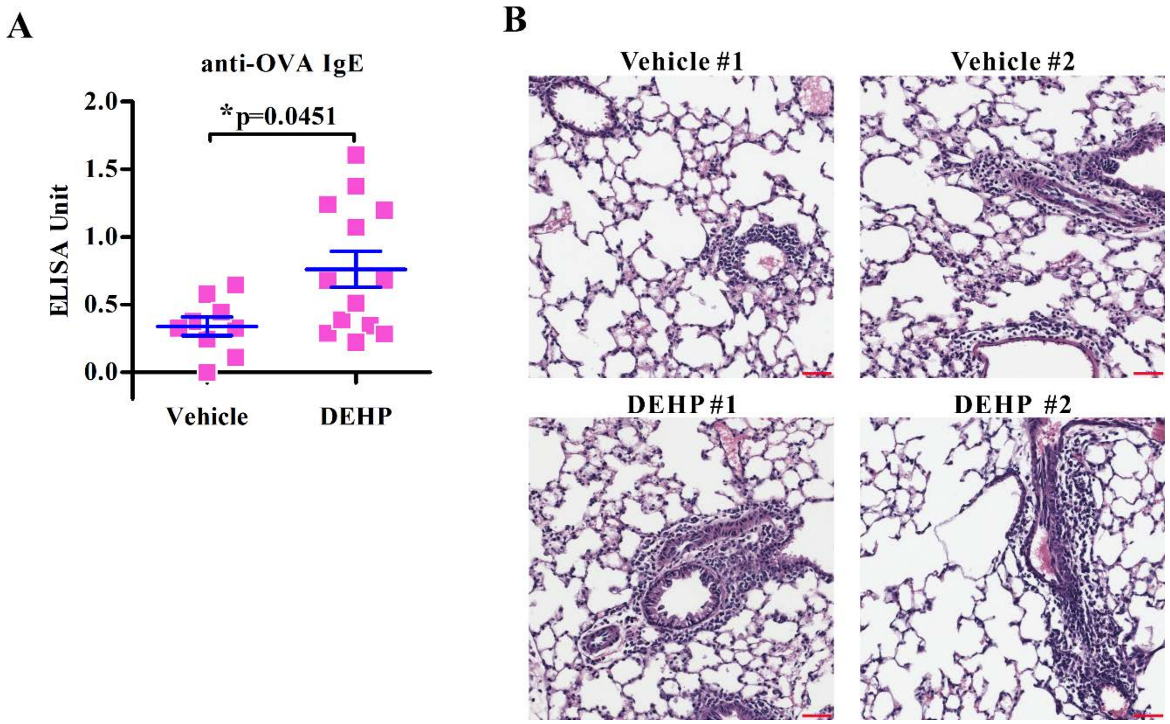

Supplementary Figure 5. F4 neonates from vehicle or DEHP-exposed F0 female mice were immunized and challenged with OVA, as described in **Supplementary Figure 1C**. **(A)** Titer of anti-OVA IgE in serum. Results are shown as mean ± SEM. $n = 9$ or 13 in the F4 offspring (3 dams each group). *p-value < 0.05 by Mann-Whitney U test. The number of offspring (n) are pooled from three independent breeding. **(B)** Representative lung sections from two OVA-immunized F4 offspring each group stained with hematoxylin and eosin. Scale bars represent 50 µm.

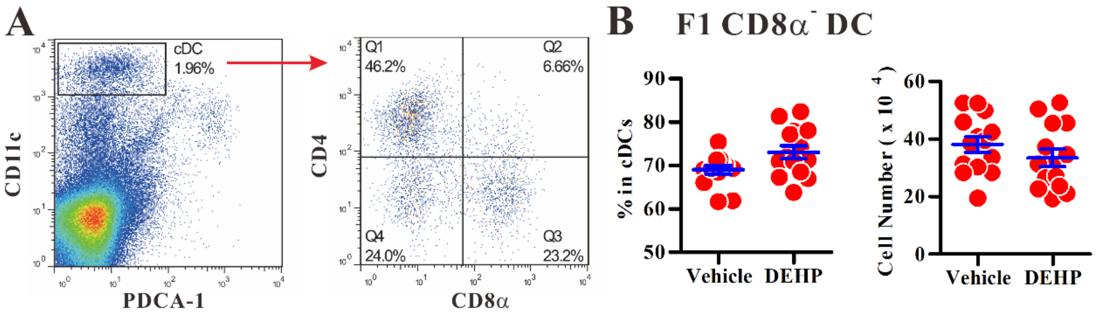

Supplementary Figure 6. Altered homeostasis of cDC following ancestral DEHP exposure in immunized F1 offspring. **(A)** Representative dot plots of viable splenic cDCs ($CD11c^{high}PDCA\text{-}1^{-}$) (left) and DC subsets (right) gated on cDCs in OVA-immunized F1 pups. **(B)** The percentage (left) and number (right) of $CD8\alpha^{-}$ DCs ($CD4^{+}CD8\alpha^{-}$ and $CD4^{-}CD8\alpha^{-}$) gated on splenic cDCs from immunized F1 offspring. Results are shown as mean ± SEM. *n* = 14 from 4 dams in each group. The number of offspring (*n*) are pooled from four independent breeding.

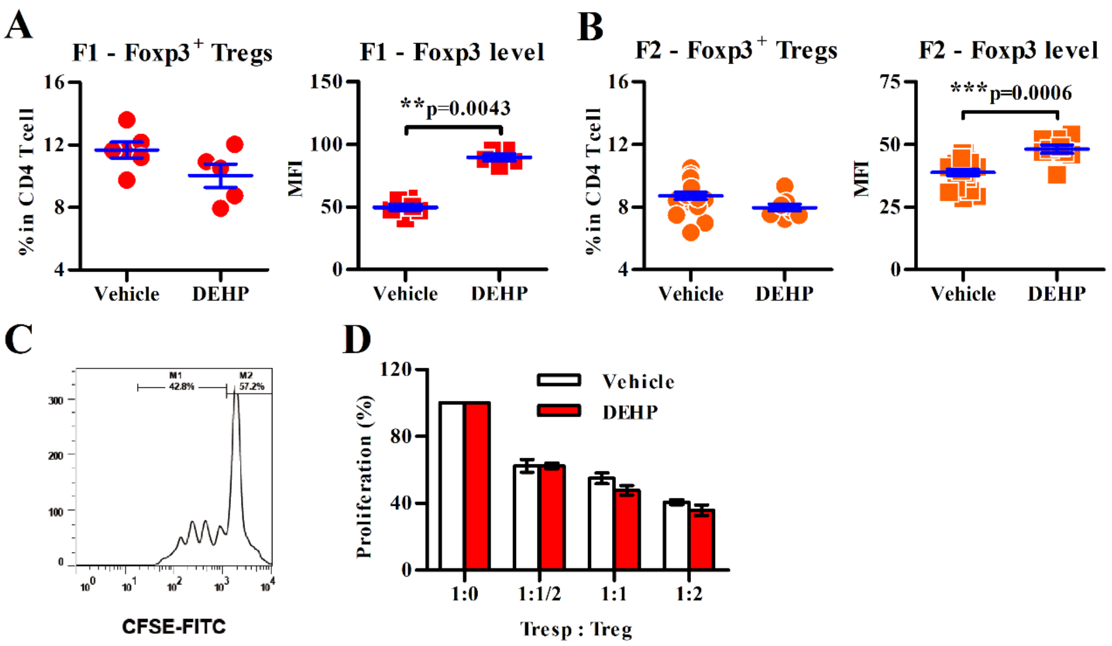

Supplementary Figure 7. Suppressive function of $CD4^+CD25^+$ Treg cells in offspring. F1 or F2 neonates from vehicle or DEHP-exposed female F0 mice were immunized with OVA as described in **Supplementary Figure 1C**. The percentages of $CD4^+CD25^+Foxp3^+$ Tregs and expression level (MFI) ratios of Foxp3 in $CD4^+CD25^+$ T cells were analyzed in F1 **(A)** or F2 **(B)** splenocytes using flow cytometry. Representative data from three independent experiments. Results are shown as mean ± SEM. * p-value < 0.05 by Mann-Whitney U test. n = 5-19 animals each group. **(C)** CFSE-labeled $CD4^+$ T cells (Tresp) from naïve female C57BL/6 mice were co-cultured with $CD4^+CD25^+$ Tregs from female F1 mice at different ratios in the presence of anti-CD3/anti-CD28 coated beads. **(D)** The percentages of proliferation (M1 at different ratios versus M1 at Tresp: Treg 1: 0) are presented as mean ± SD. n = 4 or 5 animals each group. Representative data from two independent experiments. Results are shown as mean ± SEM.

SUPPLEMENTARY REFERENCE

1. Lin L. C., Wang S. L., Chang Y. C., Huang P. C., Cheng J. T., Su P. H., et al., Associations between maternal phthalate exposure and cord sex hormones in human infants. *Chemosphere* (2011) 83:1192-9. doi: 10.1016/j.chemosphere.2010.12.079
